# Biomarkers for Immunotherapy in Driver-Gene-Negative Advanced NSCLC

**DOI:** 10.3390/ijms241914521

**Published:** 2023-09-25

**Authors:** Yiyi Huang, Yi-Fung Chau, Hua Bai, Xinyu Wu, Jianchun Duan

**Affiliations:** CAMS Key Laboratory of Translational Research on Lung Cancer, State Key Laboratory of Molecular Oncology, Department of Medical Oncology, National Cancer Center/National Clinical Research Center for Cancer/Cancer Hospital, Chinese Academy of Medical Sciences, Peking Union Medical College, Beijing 100021, China; hydrogen_11@163.com (Y.H.); yifeng_1997@126.com (Y.-F.C.); 13810095769@163.com (H.B.); wxy_9707@163.com (X.W.)

**Keywords:** PD-L1, immuno-monotherapy, immunotherapy combined with chemotherapy, predictive biomarkers, non-small cell lung cancer

## Abstract

Outcome improvement in patients with driver-gene-negative advanced non-small cell lung cancer (NSCLC) has been significantly enhanced through targeting the immune system, specifically the PD-L1/PD-1 axis. Nevertheless, only a subset of patients with advanced NSCLC may derive benefits from immuno-monotherapy or immunotherapy combined with chemotherapy. Hence, in order to identify patients who will gain the maximum advantage from immunotherapy, it is crucial to investigate predictive biomarkers. This review provides a summary of the currently identified biomarkers associated with the extent of benefit from immuno-monotherapy or immunotherapy combined with chemotherapy in patients with advanced NSCLC. These biomarkers can be categorized into three groups: tumor-related, tumor-microenvironment-related, and host-factor-related.Tumor-related factors include PD-L1 expression, tumor mutational burden and specific genetic mutations, while tumor-microenvironment-related factors include extracellular vesicles and T-cell receptors, and host-related factors include systemic inflammation, circulating fatty acid profile, and the microbiome.

## 1. Introduction

**Figure 1:**
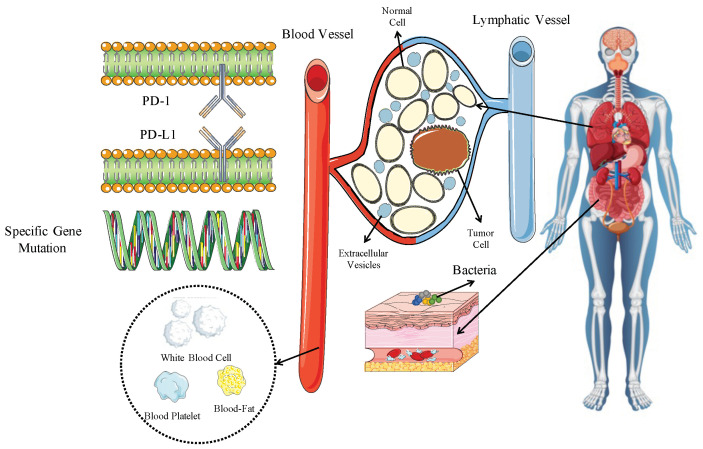
Potential biomarkers of the response to ICIs.

Immunotherapy has revolutionized the treatment of advanced cancer, specifically non-small cell lung cancer (NSCLC), by leveraging the immune system’s potential to eliminate cancer cells [1]. Impaired immune surveillance plays a crucial role in all stages of tumorigenesis, encompassing initiation, progression, and metastasis. This phenomenon arises from tumor cells evading immune surveillance, ultimately enabling the abnormal cells to proliferate and metastasize, leading to tumorigenesis [2]. Immune checkpoint inhibitors (ICIs) have emerged as a promising therapeutic approach to restore immune surveillance in cancer patients. Numerous studies and clinical trials have exhibited the efficacy of ICIs in treating lung cancer, particularly in patients with driver-gene-negative advanced NSCLC [3]. Despite the notable success of ICIs, a significant proportion of patients do not respond to the treatment, and some may even experience hyperprogression. Hence, there is an urgent need to identify predictive biomarkers capable of accurately identifying patients most likely to respond to ICIs. In this review, we classify biomarkers into three categories based on the stepwise nature of the immune response process: tumor-related, tumor-microenvironment (TME)-related, and host-related (Figure 1). Our aim is to identify suitable biomarkers for the prediction or selection of patients suitable for immunotherapy and to further evaluate the similarities and differences between biomarkers for immunotherapy alone and immunotherapy in combination with chemotherapy.

## 2. Tumor-Related

### 2.1. PD-L1

PD-L1 (programmed death-ligand 1) is expressed on tumor cells and can bind PD-1 on T cells to inhibit T-cell activity. As the center of anti-tumor action in immunotherapies, PD-L1 expression represented by immunohistochemistry (IHC), whose interpretation typically focuses on the ratio of tumor cells (TC) with membranous staining, serves as a key biomarker in predicting the efficacy of ICIs. PD-L1 testing is recommended in advanced or metastatic NSCLC [4]. Recommendations for systemic therapies takes PD-L1 expression into account.

PD-L1 expression was assessed using different antibodies and assays in different clinical trials, whose definition of positive or negative results were based on trials and may be unique to the ICI. For example, with pembrolizumab, researchers used anti-PD-L1 Dako clone 22C3 and defined the positive threshold of TC staining as ≥1% and ≥50% [5,6]. It is generally agreed that PD-L1 expression levels across different platforms are in high concordance [7,8] and it is not recommended to conduct multiple IHC tests in individuals [4]. Therefore, we discuss PD-L1 expression as one biomarker despite the existence of different platforms or antibodies.

PD-L1 expression seems to be a good biomarker in first-line PD-1/L1-inhibitor monotherapy. In practice, according to the NCCN guidelines, single agent ICIs are recommended for first-line treatment in patients with PD-L1 expression levels of ≥50%, with only pembrolizumab also recommended in patients with PD-L1 expression levels of ≥1% [9]. In the KEYNOTE−024 [10], KEYNOTE-042 [11], IMpower110 [12] and EMPOWER-Lung 1 [13] trials, patients with PD-L1 expression levels of ≥50% benefited from pembrolizumab, atezolizumab, and cemiplimab, respectively. While in subgroups of PD-L1 expression ≥1%, only pembrolizumab demonstrated a prolonged OS (HR = 0.81, 95% CI: 0.71–0.93) [11], compared to negative results in IMpower110 [12] (HR = 1.04, 95% CI: 0.76–1.44) (detailed in Table 1).

A combination of chemotherapy and immunotherapy confers clinical benefit regardless of PD-L1 expression [4]. Multiple trials, including CameL-sq [16,17], IMpower132 [18], KEYNOTE-189 [19], and GEMSTONE-302 [20], exhibited that the OS or PFS of subgroups with PD-L1 expression < 1% can be significantly prolonged (detailed in Table 2).

In a second-line setting, the efficacy of ICI mono-treatment seems less dependent on the expression of PD-L1. Apart from KEYNOTE-010 [21], which only recruited patients with PD-L1 expression ≥1%, other trials demonstrated the benefits of second-line immuno-monotherapy regardless of PD-L1 expression, including the OAK [22], POPLAR [23], Checkmate-017 and Checkmate-057 [24], Checkmate-078 [25], and RATIONALE-303 [26] trials. However, a tendency of more benefit in patients with higher expression of PD-L1 can be observed. In KEYNOTE-010, a trend of longer OS was observed in PD-L1 TPS ≥50% versus TPS >1% [5], especially in newly collected samples [27]. Notably, the HR for OS and PFS was significantly different between subgroups of PD-L1 expression with the cutoff of 5% and 10% in CheckMate 057 [28] (detailed in Table 3). Meanwhile, in second-line combined therapy of ICI and chemotherapy, the predictive role of PD-L1 remains unclear due to a small sample, as presented in Table 4.

**Table 2:** Summary of HR for mOS or mPFS based on PD-L1 expression in first-line immunotherapy combined with chemotherapy.

Clinical Trial	Pathological Type	Treatment Arms	HR for mOS (95% CI) by PD-L1 (%)	HR for PFS (95% CI) by PD-L1 (%)
**Squamous**				
IMpower131 [29]	SQ	Atezolizumab + Nab-paclitaxel + carboplatin vs. Nab-paclitaxel + carboplatin	TC/IC 3 HR = 0.48, 95% CI 0.29–0.81	TC/IC 3 HR = 0.41, 95% CI 0.25–0.68
			TC/IC 1/2 HR = 1.08, 95% CI 0.81–1.45	TC/IC 1/2 HR = 0.70, 95% CI 0.54–0.91
			TC/IC 0 HR = 0.87, 95% CI 0.67–1.13	TC/IC 0 HR = 0.82, 95% CI 0.65–1.04
KEYNOTE-407 [30]	SQ	Pembrolizumab + chemotherapy vs. chemotherapy	≥50% HR = 0.68, 95% CI 0.47–0.97	≥50% HR = 0.48, 95% CI 0.33–0.69
			1–49% HR = 0.61, 95% CI 0.45–0.83	1–49% HR = 0.6, 95% CI 0.45–0.81
			<1% HR = 0.83, 95% CI 0.61–1.13	<1% HR = 0.7, 95% CI 0.52–0.95
RATIONALE-307 [31]	SQ	Tislelizumab + paclitaxel/nab-paclitaxel and carboplatin vs. paclitaxel and carboplatin	NA	≥50% $HR{\;}^{a}$ = 0.50, 95% CI 0.28–0.89
				1–49% $HR{\;}^{a}$ = 0.44, 95% CI 0.22–0.87
				<1% $HR{\;}^{a}$ = 0.64, 95% CI 0.37–1.10
				≥50% $HR{\;}^{b}$ = 0.43, 95% CI 0.23–0.78
				1–49% $HR{\;}^{b}$ = 0.31, 95% CI 0.15–0.66
				<1% $HR{\;}^{b}$ = 0.69, 95% CI 0.41–1.18
CameL-sq [16,17]	SQ	Camrelizumab + chemotherapy vs. chemotherapy	≥50% HR = 0.48, 95% CI 0.21–1.12	≥50% HR = 0.30, 95% CI 0.17–0.55
			1–49% HR 0.6, 95% CI 0.27–1.0	1–49% HR 0.6, 95% CI 0.20–0.51
			<1% HR 0.7, 95% CI 0.41–0.94	<1% HR 0.7, 95% CI 0.35–0.68
ORIENT-12 [32]	SQ	Sintilimab + chemotherapy vs. chemotherapy	NA	≥50% HR = 0.458, 95% CI 0.302–0.695
				1–49% HR 0.620, 95% CI 0.408–0.941
				<1% HR 0.548, 95% CI 0.368–0.815
**Non-squamous**				
IMpower130 [33]	NSQ	Atezolizumab + Nab-paclitaxel + carboplatin vs. Nab-paclitaxel + carboplatin	TC/IC 3 HR = 0.84, 95% CI 0.51–1.39	NA
			TC/IC 1/2 HR = 0.7, 95% CI 0.45–1.08	
			TC/IC 0 HR = 0.81, 95% CI 0.61–1.08	
IMpower132 [18]	NSQ	Atezolizumab + Carboplatin/ cisplatin + pemetrexed vs. Carboplatin/cisplatin + pemetrexed	TC/IC 3 HR = 0.73, 95% CI 0.31–1.73	NA
			TC/IC 1/2 HR = 1.18, 95% CI 0.80–1.76	
			TC/IC 0 HR = 0.67, 95% CI 0.46–0.96	
KEYNOTE-189 [19]	NSQ	Pembrolizumab + chemotherapy vs. chemotherapy	≥50% HR = 0.59, 95% CI 0.40–0.86	
			1–49% HR = 0.66, 95% CI 0.46–0.96	
			<1% HR = 0.51, 95% CI 0.36–0.71	
RATIONALE-304 [34]	NSQ	Tislelizumab + chemotherapy vs. chemotherapy	NA	≥50% HR = 0.336, 95% CI 0.185–0.611
				1–49% HR = 1.095, 95% CI 0.526–2.277
				<1% HR = 0.733, 95% CI 0.456–1.179
CameL [35]	NSQ	Camrelizumab + chemotherapy vs. chemotherapy	NA	≥50% HR = 0.39, 95% CI 0.14–0.99
				1–49% HR = 0.62, 95% CI 0.40–0.94
				<1% HR = 0.76, 95% CI 0.45–1.26
ORIENT 11 [36]	NSQ	Sintilimab + chemotherapy vs. chemotherapy	≥1% HR = 0.56, 95% CI 0.40–0.77	NA
			<1% HR = 0.75, 95% CI 0.48–1.19	
**NSCLC**				
GEMSTONE-302 [20]	NSCLC	Sugemalimab + chemotherapy vs. chemotherapy	NA	≥50% HR = 0.41, 95% CI 0.27–0.62
				1–49% HR = 0.53, 95% CI 0.35–0.79
				<1% HR = 0.56, 95% CI 0.40–0.77
CHOICE-01 [37]	NSCLC	Toripalimab + chemotherapy vs. chemotherapy	All HR = 0.69, 95% CI 0.53–0.92	HR = 0.49; 95% CI 0.39–0.61
			≥50% HR = 0.56, 95% CI 0.36–0.86	TC ≥50% HR = 0.45; 95%CI 0.27–0.78
			1–49% HR = 0.72, 95% CI 0.48–1.07	1%≤ TC < 50% HR = 0.56; 95% CI 0.40–0.78
			<1% HR = 0.79, 95% CI 0.49–1.31	TC < 1% HR = 0.47; 95%CI 0.32–0.71
EMPOWER-Lung 3 [38]	NSCLC (EGFR/ALK/ROS1WT)	Cemiplimab + chemotherapy vs. chemotherapy	All HR = 0.65, 95% CI: 0.51–0.82	NA
			≥50% HR = 0.56, 95% CI 0.36–0.86	
			1–49% HR = 0.50, 95% CI 0.34–0.74	
			<1% HR = 0.94, 95% CI 0.62–1.42	

Note: ^*a*^ Tislelizumab plus paclitaxel and carboplatin vs. paclitaxel and carboplatin. ^*b*^ Tislelizumab plus nab-paclitaxel and carboplatin vs. paclitaxel and carboplatin. CI, confidence interval; HR, hazard ratio; IC, immune cell; ITT, intention to treat; NA, not available; NR, not reached; ORR, objective response rate; OS, overall survival; TC, tumor cell; TC/IC 0, PD-L1 expression on 0% of tumor and immune cells; TC/IC 1, PD-L1 expression of ≥1% on either tumor cells or immune cells; TC/IC 2, PD-L1 expression on 1–49% of tumor cells or on 1–10% of immune cells; TC/IC 3, PD-L1 expression on ≥50% of tumor cells or on ≥10% of immune cells; WT, wild type; NA, not available.

**Table 3:** Summary of HR for mOS or mPFS based on PD-L1 expression in second-line immuno-monotherapy.

Clinical Trial	Pathological Type	Treatment Arms	HR for mOS (95% CI) by PD-L1 (%)	HR for PFS (or mPFS) (95% CI) by PD-L1 (%)
KEYNOTE-010 [21]	NSCLC	Pembrolizumab vs. Docetaxel	TPS ≥50%: 16.9 vs. 8.2 mo, HR = 0.55 (0.44–0.69)	TPS ≥50%: 5.3 vs. 4.2 mo, HR = 0.57 (0.46–0.71)
			TPS 1–49%: HR = 0.79(0.65–0.94)	TPS ≥1%: 4.0 vs. 4.1 mo, HR = 0.84 (0.73–0.96)
			TPS ≥1%: 11.8 vs. 8.4mo, HR = 0.70 (0.61–0.80)	
OAK [22]	NSCLC	Atezolizumab vs. Docetaxel	ITT: 13.8 vs. 9.6 mo, HR 0.73 (0.62–0.87)	ITT: 2.8 vs. 4.0 mo, HR 0.95 (0.82–1.10)
			TC3 or IC3: 20.5 vs. 8.9 mo, HR 0.41(0·27–0·64)	TC3 or IC3: 4.2 vs. 3.3 mo, HR 0.63(0.43–0.91)
			TC2/3 or IC2/3: 16.3 vs. 10.8 mo, HR 0.67 (0.49–0.90)	TC2/3 or IC2/3: 4.1 vs. 3.6 mo, HR 0.76(0.58–0.99)
			TC1/2/3 or IC1/2/3: 15.7 vs. 10.3 mo, HR 0·74 (0·58–0.93)	TC1/2/3 or IC1/2/3: 2.8 vs. 4.1 mo, HR 0.91 (0·74–1.12)
			TC0 and IC0: 12.6 vs. 8.9 mo, HR 0.75 (CI 0.59–0.96)	TC0 and IC0: 2.6–4.0 mo, HR 1.00 (0.80–1.25)
POPLAR [23]	NSCLC	Atezolizumab vs. Docetaxel	TC3 or IC3: 15.5 vs. 11.1 mo, HR 0·49 (0·22–1.07)	TC3 or IC3: 7.8 vs. 3.9 mo, HR 0.60 (0.31–1.16)
			TC2/3 or IC2/3: 15.1 vs. 7.4 mo, HR 0.54 (0.33–0.89)	TC2/3 or IC2/3: 3.4 vs. 2.8 mo, HR 0.72 (0.47–1.10)
			TC1/2/3 or IC1/2/3: 15.5 vs. 9.2 mo, HR 0·59 (0·40–0·85)	TC1/2/3 or IC1/2/3: 2.8 vs. 3.0 mo, HR 0.85 (0.63–1.16)
			TC0 and IC0: 9.7 vs. 9.7 mo, HR 1.04 (0.62–1.75)	TC0 and IC0: 1.7 vs. 4.1mo, HR 1.12 (1.4–4.2)
Checkmate-017 and Checkmate-057 [24]	NSCLC	Nivolumab vs. Docetaxel	PD-L1 ≥1%: 13.4 vs. 8.5 mo, HR 0.61 (0.49–0.76)	PD-L1 ≥1%: 3.7 vs. 3.6 mo, HR = 0.66 (0.53–0.84)
			PD-L1 <1%: 9.7 vs. 7.8 mo, HR 0.76 (0.61–0.96)	PD-L1 <1%: 2.1 vs. 3.5 mo, HR 0.99 (0.78–1.26)
Checkmate-078 [25]	NSCLC	Nivolumab vs. Docetaxel	PD-L1 ≥1 %: 12.0 vs. 7.9 mo, HR 0.71 (0.54–0.95)	PD-L1 tumor expression ≥1%: 2.8 vs. 2.6 mo, HR 0.75 (0.56–0.99)
			PD-L1 <1%: 11.4 vs. 10.2 mo, HR 0.73 (0.53–1.02)	PD-L1 expression <1%: 2.9 vs. 2.8 mo, HR 0.77 (0.56–1.07)
RATIONALE-303 [26]	NSCLC	Tislelizumab vs. docetaxel	PD-L1 expression ≥25% TC: 19.3 vs. 11.5 mo, HR 0.53 (0.40–0.70)	PD-L1 expression ≥25% TC: 0.37 (0.28–0.49)
			PD-L1 expression <25% TC: 15.2 vs. 12.3 mo, HR 0.77 (0.62–0.96)	

Note: CI, confidence interval; HR, hazard ratio; IC, immune cell; ITT, intention to treat; NA, not available; NR, not reached; ORR, objective response rate; OS, overall survival; TC, tumor cell; TC/IC 0, PD-L1 expression on 0% of tumor and immune cells; TC/IC 1, PD-L1 expression of ≥1% on either tumor cells or immune cells; TC/IC 2, PD-L1 expression on 1–49% of tumor cells or on 1–10% of immune cells; TC/IC 3, PD-L1 expression on ≥50% of tumor cells or on ≥10% of immune cells; WT, wild type.

**Table 4:** Summary of HR for mOS or mPFS based on PD-L1 expression in second-line immunotherapy combined with chemotherapy.

Clinical Trial	Pathological Type	Treatment Arms	HR for mOS (95% CI) by PD-L1 (%)	HR for PFS (or mPFS) (95% CI) by PD-L1 (%)
TORG1630 [39]	NSCLC	Nivolumab vs. Nivolumab plus Docetaxel	PD-L1 ≥ 50% (*N* = 5): HR 1.03 (0.09–11.55)	NA
			PD-L1 1–49% (*N* = 23): HR 0.32 (0.10–1.07)	
			PD-L1 0% (*N* = 22): HR 0.41 (0.14–1.22)	
PROLUNG [40]	NSCLC	Pembrolizumab plus Docetaxel vs. Docetaxel	NA	PD-L1 (+) (*N* = 30): 16.8 vs. 3.9 mo, HR 0.16 (0.05–0.52)
				PD-L1 (−) (*N* = 30): 6.3 vs. 4.4 mo, HR 0.41 (0.16–1.05)

Efforts are made to discover new techniques for the assessment of PD-L1. Soluble PD-L1 was an associated prognosis in a cohort of 128 patients who received ICIs [41]. Blood-based dynamic changes in PD-L1 expression in tumor-associated cells (TACs) were identified as a biomarker for ICI efficacy in a prospective study (*N* = 82). Increased PD-L1 expression in TACs after ICI treatment was associated with significant prolonged PFS (HR 3.49, 95% CI: 1.5–8.3) and OS (HR 3.058, 95% CI: 1.2–7.9) [42].

The general trend towards a better efficacy in patients with higher expression of PD-L1 can be observed in studies. However, PD-L1 was mainly studied as a biomarker in the subgroup analysis of clinical trials, whose exploratory nature and small number of patients in the subgroups limit the conclusion. Meta-analysis provides evidence for different combination treatment in subsets of PD-L1 expression [43].

### 2.2. Tumor Mutation Burden (TMB)

#### 2.2.1. Tissue TMB (tTMB)

Tumor mutation burden (TMB) refers to the number of somatic mutations present in the tumor genome, excluding germline mutations [44]. It is quantified as the total number of identified somatic gene coding errors, base substitutions, and gene insertion or deletion errors per million bases. TMB has gained increasing attention as a potential alternative measure [45]. A meta-analysis conducted in 2019 explored the relationship between TMB and the outcomes of patients treated with PD-1/PD-L1 inhibitors, revealing a positive correlation between TMB and the efficacy of immunotherapy [46]. Recent studies, such as KEYNOTE-042, demonstrated that tissue-TMB (tTMB) can serve as a predictive biomarker for pembrolizumab monotherapy in patients with advanced/metastatic PD-L1 tumor proportion scores of ≥1% NSCLC (p<0.001), with a tTMB cut-off of ≥175 mutations/exome [47]. The association between tTMB and clinical outcomes has also been validated in patients treated with pembrolizumab monotherapy [48], atezolizumab monotherapy [49,50], nivolumab monotherapy [14], nivolumab plus ipilimumab [51,52,53], and durvalumab plus tremelimumab [15]. Liquid biopsy, which involves analyzing biomarkers in body fluids, has the potential to reduce biases associated with tumor heterogeneity present in tissue biopsies [54]. Studies utilizing liquid biopsy have shown a positive correlation between blood-TMB (bTMB) and tTMB, suggesting that bTMB could serve as a promising prognostic biomarker for NSCLC patients receiving immunotherapy [55,56]. The use of liquid biopsy in the context of atezolizumab treatment in second-line and higher NSCLC patients has identified bTMB as a means to identify patients who experience clinically significant improvements in PFS, with high bTMB correlating with a PFS benefit in those treated with atezolizumab monotherapy. These findings were further supported by the 2L NSCLC Phase III OAK and Phase II POPLAR studies [50,57]. In addition to second-line NSCLC patients, bTMB has also demonstrated predictive significance for first-line patients receiving atezolizumab monotherapy, as shown in the B-F1RST primary analysis. In patients with high bTMB, the ORR was 28.6% (8/28) vs. 4.4% (4/91) in those with low bTMB, and the median PFS was 4.6 months vs. 3.7 months (HR = 0.66, 90% CI: 0.42–1.02, p=0.12). Moreover, the median OS was not estimable (NE) vs. 13.1 months in patients with high vs. low bTMB, respectively (HR = 0.77, 90% CI 0.41–1.43, p=0.48) [58]. This shows that there is a tendency to benefit, but the *p*-value is not statistically significant.

TMB has been shown to have predictive value not only in immunotherapy monotherapy but also in combination with chemotherapy. This predictive role was confirmed in the CHOICE-01 study. It demonstrated that TMB-high patients in the toripalimab combination group had a higher objective response rate (72.7% vs. 46.7%) compared to the chemotherapy-alone group, which aligns with the response rates observed in the intention-to-treat population (65.7% vs. 46.2%). Moreover, TMB-high patients in the toripalimab combination group had significantly longer median PFS compared to those in the chemotherapy-alone group (13.1 months vs. 5.5 months; HR = 0.34; 95% CI: 0.21–0.54; interaction p=0.026), while no significant difference in OS was observed between the two TMB subgroups (interaction p=0.9962) [37]. Similarly, the POSEIDON study revealed that tremelimumab (T) + durvalumab (D) + chemotherapy (CT) demonstrated longer median OS in both the bTMB ≥ 20 mut/Mb and < 20 mut/Mb subgroups, with higher benefit observed in the bTMB-high group. For patients with bTMB ≥ 20 mut/Mb, median OS was 13.5 months with T+D+CT vs. 10.3 months with CT (unstratified HR = 0.61; 95% CI: 0.42–0.88), whereas for patients with bTMB < 20 mut/Mb, median OS was 12.6 months vs. 10.9 months (unstratified HR = 0.79; 95% CI: 0.63–0.99) [59]. PFS and ORR exhibited similar trends to OS. However, the prospective phase III BFAST trial concluded that bTMB at a cutoff of 16 mut/exome was not predictive of clinical outcomes with atezolizumab in previously untreated metastatic NSCLC (HR for PFS, 0.77; 95% CI: 0.59–1.00; HR for OS, 0.87; 95% CI: 0.64–1.17) [60]. However, in KEYNOTE-189, TMB as a continuous variable was not significantly associated with OS, PFS, or ORR for pembrolizumab plus chemotherapy (one-sided *p* = 0.174, 0.075 and 0.072, respectively) or placebo plus chemotherapy (two-sided *p* = 0.856, 0.055 and 0.434, respectively). The OS benefit of pembrolizumab plus chemotherapy was similar in the high-TMB subgroup and the low-TMB subgroup [61]. This phenomenon was also observed in KEYNOTE-407, which may indicate that tTMB was not significantly associated with the effectiveness of pembrolizumab combined with platinum-based chemotherapy or chemotherapy alone as 1L therapy for metastatic NSCLC, regardless of histology [62]. Overall, the correlation between TMB and the prognosis of immunotherapy requires further investigation.

#### 2.2.2. Blood TMB (bTMB)

Tissue TMB may be challenging to obtain, especially in advanced NSCLC. Correlated with tTMB, blood TMB (bTMB) was identified as a biomarker for PFS but not OS in the POPLAR cohort. The HR that favored atezolizumab in the population with bTMB ≥ 16 mut/Mb was 0.65 (95% CI 0.47–0.92), compared to 0.98 (95% CI 0.80–1.20) in patients with bTMB < 16 mut/Mb. This was validated in the OAK cohort [50]. Due to the second-line population of the POPLAR and OAK studies, DNA-damaging agents prior to blood sampling and longer storage time may lead to discordance between bTMB and tTMB. The prospective phase 2 B-F1RST trial [63] aimed to validate bTMB in first-line treatment, based on the IMpower 110 trial. However, the study failed to meet the biomarker endpoint in PFS, with mPFS at 5 vs. 3.5 months in patients with bTMB ≥ 16 mut/Mb and < 16 mut/Mb group (HR = 0.80, 90% CI 0.54–1.18), but ORR was significantly improved with bTMB ≥ 16 mut/Mb. The role that bTMB plays in the prediction of ICIs has not yet been clarified, nor has its best cut-off. In contrary to previous assumptions that higher TMB correlated with better ICI efficacy, a study utilizing data from the OAK and POPLAR trials found a non-linear correlation between bTMB and ICI efficacy. Low bTMB of ≤7 mut/Mb and high bTMB of ≥14 mut/Mb were identified as conferring a better prognosis than medium bTMB of 8–13 mut/Mb [64].

A combination of bTMB with other biomarkers is one way to improve its prediction efficacy. In the cohorts of POPLAR (*N* = 211), OAK (*N* = 462) studies and a retrospective cohort (*N* = 64), bTMB was not associated with OS in the second-line immunotherapy. However, with the adjustment of maximum allele frequency (AF), which reflects blood ctDNA, low AF-bTMB-H, whose cut-off point was 12, was associated with favorable OS (HR = 0.70, 95% CI: 0.52–0.95) and PFS (HR = 0.62, 95% CI: 0.47– 0.80). The predictive efficacy was further validated with the retrospective cohort (OS: HR = 0.20, 95% CI: 0.05–0.84; PFS: HR = 0.30, 95% CI: 0.13–0.70) [57,65]. This biomarker was further validated with the cohorts of the Geneplus Cancer Genome Database and other retrospective cohorts [66].

The innovative computation of blood biopsy sequencing data generated blood Intratumor heterogeneity (bITH) as a predictive biomarker for immunotherapy in the OAK and POPLAR cohorts, which is more effective than bTMB (OS: HR = 0.56, 95% CI: 0.41–0.77 vs. HR = 0.94, 95% CI: 0.68–1.29; PFS: HR = 0.72, 95% CI: 0.55–0.93 vs. HR = 1.18, 95% CI: 0.89–1.56). This is further validated in an independent retrospective cohort (*N* = 42) [67]. Another study introduced ctDNA-adjusted bTMB as significant biomarkers for both OS (*p* = 0.016) and PFS (*p* = 0.002) in the POPLAR and OAK cohorts, validated by independent cohorts (*N* = 47 and *N* = 44) [68].

However, there are trials which did not observe a correlation between efficacy and tTMB or bTMB [51,69]. The NCCN guidelines have removed TMB as an immune biomarker for patients with metastatic NSCLC. To date, TMB is only a measure of phenotype, reflecting overall tumor burden, with no direct link with neoantigen load or antigenicity or antigen-presenting pathway. To better utilize TMB as biomarkers, further studies into its biological role and consequences are still pending.

### 2.3. Specific Genetic Mutations

It is generally agreed that patients with driver-gene mutations have limited benefits from immunotherapy. Consequently, we discuss the biomarkers for immunotherapy in advanced NSCLC patients without driver-gene mutations. Here, we discuss gene mutations that are not generally included in driver genes (EGFR mutations, ALK alterations and ROS1 mutations, etc.).

The Serine/threonine kinase 11 (STK11) protein is involved in the regulation of lipid, glucose, and cholesterol metabolism by activating AMP-activated protein kinases [70]. Kelch-like ECH-associated protein 1 (KEAP1) functions as an inhibitor of nuclear factor-erythroid 2-related factor 2 (NRF2), which controls the expression of detoxification genes and cytoprotective enzymes crucial for metabolism, oxidative stress, inflammation, and the cellular response to anticancer treatments [71]. Loss of this protein allows cancer cells to proliferate and reprogram themselves metabolically, enabling them to withstand chemotherapy, radiotherapy, and immunotherapy. Inactivation of this protein leads to reduced levels of CD8+ T lymphocytes in both human and mouse models, indicating compromised immune surveillance of tumors [72]. Several studies have proposed that mutations in STK11 and KEAP1 contribute to resistance to immune checkpoint inhibitors. Papillon-Cavanagh et al. evaluated the impact of STK11 and KEAP1 mutations in NSCLC samples on the response to various treatments, including PD-1/PD-L1 inhibitors, EGFR inhibitors, vascular endothelial growth factor inhibitors, platinum chemotherapy, and chemotherapy alone [73]. Among the 2276 cases analyzed, mutations in STK11, KEAP1, and co-occurring mutations in both genes were observed in 20%, 20%, and 10% of cases, respectively. Furthermore, 75.8% of samples with STK11 and/or KEAP1 mutations showed negative PD-L1 staining, in contrast to 60.8% of samples with wild-type STK11 and KEAP1 (p<0.001). Real-world data indicated that patients with co-existing STK11 and KEAP1 mutations treated with PD-1/PD-L1 inhibitors, anti-VEGF, EGFR inhibitors, platinum doublets, or single-agent chemotherapy had shorter mPFS. More specifically, co-mutations of KEAP1 and STK11 were associated with poorer mPFS than mutations in KEAP1 or STK11 alone in patients treated with anti-PD-1/PD-L1 therapy. Therefore, the co-occurrence of STK11 and KEAP1 mutations serves as a predictive factor for systemic therapies, including immunotherapy.

In the CHOICE-01 study, researchers found that patients harboring SMARCA4 mutations, particularly in the non-squamous subgroup (*n* = 33), achieved significantly better PFS in the toripalimab-combination arm than in the chemotherapy-alone arm (median PFS: 9.9 vs. 2.9 months, Data Supplement) [37]. However, in patients with squamous cell carcinoma harboring SMARCA4 mutations (*n* = 21), there was a correlation between worse PFS with the toripalimab-combination arm compared to the chemotherapy-alone arm (median PFS 4.2 versus 8.2 months), suggesting a potential lack of efficacy in these patients. They also found the PI3K-Akt-mTOR pathway with common genes such as COL3A1, COL6A3, FLT1, FLNC, HGF, IRS1, IRS2, ITGA4, ITGA8, and KDR emerged as one of the most enriched pathways for treatment response. Patients carrying mutations in this pathway showed significantly better PFS and OS when treated with toripalimab combined with chemotherapy compared to chemotherapy alone. Additionally, patients in the toripalimab-combination arm also had a favorable PFS if they had alterations in genes downstream of the IL-7 signaling pathway (HGF, IRS1, IRS2, and SMARCA4) or in the SWI/SNF chromatin remodeling complex (SMARCA4, SMARCA2, and PBRM1). These results were further validated using three publicly available NSCLC data sets in which patients were treated with immunotherapies. Specifically, the validation sets confirmed that PI3K-Akt-alteration patients had significantly better PFS than wild-type patients.

In the OAK and POPLAR cohorts, several gene mutations were identified as biomarkers for ICI efficacy. In patients with STK11 or KEAP1 mutations, atezolizumab yielded benefits in mOS compared to docetaxel (7.3 vs. 5.8 mo, adjusted HR 0.70; 95% CI 0.49–0.99), especially in SKmut (STK11- or KEAP1-mutated) patients with FAT3 mutation (HR = 0.06, 95% CI 0.01–0.60) [74]. EPHA mutation was significantly correlated with worse efficacy (*p* = 0.0186) [75], while PALB2 mutation did not correlate with efficacy of immunotherapy (HR = 1.1, *p* = 0.75) in the POPLAR and OAK cohorts [76]. Consistent with previous findings, a 5-genomic mutation signature composed of CREBBP, KEAP1, RAF1, STK11, and TP53 mutations was discovered to be more significantly correlated with OS than bTMB score and PD-L1 [77]. Similarly, a blood-based genomic mutation signature (bGMS) was trained with the OAK cohort (OS 7.9 vs. 19.9 mo, *p* < 0.0001; PFS 1.7 vs. 4 mo, *p* = 0.011) and validated with the POPLAR cohort (OS 8.4 vs. 18.6 mo, *p* = 0.0019; PFS 1.5 vs. 4.4 mo, *p* = 0.013). In POPLAR/OAK and three other cohorts, NOTCH 1/2/3 mutation was correlated with better outcomes in ICI (PFS: HR = 0.61 (95% CI 0.46–0.81; OS: HR = 0.56 (95% CI 0.32–0.96), especially in deleterious NOTCH mutation [78].

Further analysis of genes related to immunotherapy efficacy focused on the MHC class II pathway. An updated analysis of ORIENT-11 [79] revealed a significant correlation between survival in immune-related pathways and antigen-presentation pathway, especially the MHC class II pathway. Pathway enrichment analysis showed that most genes associated with PFS were enriched in immune-related pathways, and functionally narrowed down to antigen presentation: HLA-DMB, HLA-DOA, DLA-DPB1, and HLA-DMA, which are all components of the MHC class II complex. They also found that both PFS (HR = 0.32, *p* < 0.0001) and OS (HR = 0.36, *p* = 0.0005) favored patients with high MHC-class-II-related gene expression in the immunotherapy-combination group, while in chemotherapy group, clinical outcomes were comparable. In addition, a strong correlation was observed between longer PFS and high MHC class-II expression regardless of PD-L1 expression.

## 3. Tumor Microenvironment (TME)-Related Biomarkers

### 3.1. Biomarkers in Extracellular Vesicles (EVs)

Exosomes are small membrane vesicles measuring 30–150 nm in diameter that are released into the extracellular matrix through fusion and can be secreted by various cell types, including cancer cells. They are found in several bodily fluids, such as plasma, saliva, urine, and pleural effusions [80]. Tumor-derived exosomes have a significant impact on tumors as they facilitate the transfer of functional proteins, mRNAs, or lncRNAs, thus influencing the local and systemic microenvironment [81]. In a study conducted in China, differences were observed in the expression profiles of plasma-derived exosomal lncRNAs and mRNAs between responders and non-responders to nivolumab immunotherapy [82]. The lnc-ZFP3-3-TAF1-CCNB1 pair and IL6R were identified as potential key factors for predicting immunotherapy effectiveness. Moreover, research suggests that the expression levels of specific substances in EVs are associated with the efficacy of ICIs. PANTANO F., et al. conducted a comprehensive analysis of EV-associated miRNAs produced by cancer cells and identified EV-miR-625-5p as a novel independent biomarker of response and survival in patients with NSCLC treated with ICIs, particularly in those with PD-L1 expression ≥50%. The abundance of EV-miR-625-5p was correlated with PD-L1 expression and significantly associated with response rate by Response Evaluation Criteria In Solid Tumors (p=0.0366) and overall survival (p=0.0031). Thus, EV-miR-625-5p has the potential to identify patients with improved survival outcomes [83]. Other studies investigated cytokine levels in EVs [84].

Cytokines can be selectively incorporated into EVs in response to specific stimuli, which protects them from degradation during circulation and facilitates their targeted release to specific cells, thereby regulating EV tropism. Transforming growth factor-β (TGF-β) is an immunosuppressive cytokine that plays a critical role in tumor immune evasion, therapy resistance, and metastasis [85]. Moreover, TGF-β is closely associated with immune regulation and tumor immune escape by exerting direct and indirect immunosuppressive activities. Evidence has indicated that a high expression of TGF-β in EVs is associated with poor response to ICIs, as well as shorter progression-free survival and overall survival [86]. These above results suggest that the concentration of certain substances in extracellular vesicles or mRNA expression, etc. may be potentially reliable biomarkers for the prediction of efficacy in immunotherapy.

### 3.2. Roles of T-Cell Receptors(TCR) in Prediction

Since activation of the immune response against tumor cells involves recognition of neoantigen peptides by clonally proliferating TCR [87], TCR-based biomarkers may be predictive of response to ICIs. A study by Jiefei Han et al. sequenced the complementarity-determining region 3 of the TCRβ chains isolated from PD-1+ CD8+ T cells to investigate its value in predicting response to anti-PD-1/PD-L1 therapy in NSCLC patients. The result showed that patients with high PD-1+ CD8+ TCR diversity prior to receiving ICIs showed a better response to ICIs and a longer PFS compared to patients with low diversity [6.4 months vs. 2.5 months, HR = 0.39; 95% CI: 0.17–0.94; *p* = 0.021]. In addition, patients with increased PD-1+ CD8+ TCR clonality after receiving ICIs had longer PFS (7.3 months vs. 2.6 months, HR = 0.26; 95% CI: 0.08–0.86; *p* = 0.002) than those with decreased clonality [88]. In conclusion, peripheral blood PD-1+ CD8+ T-cell TCR diversity and clonality may non-invasively predict patient response to ICIs and survival in NSCLC.

## 4. Host-Related

### 4.1. Biomarkers Relating to Systemic Inflammation

The systemic inflammatory response is also involved in the response to ICIs. By examining peripheral blood components such as white cell count (WCC), neutrophil count (NC), lymphocyte count (LC), platelet count, serum albumin, C-reactive protein (CRP), and lactate dehydrogenase (LDH), clinicians can more accurately stratify patients who would benefit from ICI treatment [89]. STARES M, et al. created The Scottish Inflammatory Prognostic Score (SIPS) to predict prognosis. SIPS assigns 1 point each for albumin <35 g/L and neutrophil count $>7.5\times 10^{9}$ /L to give a three-tier categorical score. It predicted PFS (HR = 2.06, 95% CI: 1.68–2.52, p<0.001) and OS (HR = 2.33, 95% CI: 1.86–2.92, p<0.001), and stratified PFS from 2.5 months for SIPS2, to 8.7 months for SIPS1, and to 17.9 months for SIPS0 (p<0.001) and OS from 5.1 months for SIPS2, to 12.4 months for SIPS1, and to 28.7 months for SIPS0 (p<0.001). The relative risk of death before 6 months was 2.96 (95% CI: 1.98–4.42) in patients with SIPS2 compared to those with SIPS0-1 (p<0.001) [90]. Other research explores the connection between inflammatory biomarkers and the efficacy of predicting treatment response to ICIs. This research shows that patients with a high post-treatment neutrophil-to-lymphocyte ratio (NLR) (≥5) had shorter PFS (HR = 1.1, p<0.001) and shorter OS (HR = 1.2, p<0.001). Additionally, patients with a high post-treatment platelet-to-lymphocyte ratio (PLR) (≥170) had significantly shorter PFS (HR = 1.0, p<0.001) and OS (HR = 0.9, p<0.001). Regarding the Lung Immune Prognostic Index (LIPI), researchers found a proportional relationship between LIPI status and prognosis, indicating that a better LIPI status leads to longer PFS and OS in ICI therapy. A favorable post-treatment GPS (GPS 0–2) was also associated with improved PFS (p<0.009) and OS (p<0.064) [91].

Cytokines, which are soluble proteins secreted by immune cells [84,92], were observed to be elevated in concentrations in individuals with tumors. Cytokines are well-known regulators of immune activity that can recruit immune cells to the TME and promote the expression of certain immune checkpoint molecules in the process of antitumor activities [93]. Circulating cytokine concentrations in the blood are easily detectable, suggesting their potential as predictive biomarkers for responses to ICIs. Researchers found that individuals with NSCLC with a low baseline concentration of IL-6 in plasma specimens or tumor tissues could derive greater benefit from ICIs [94]. This may be explained by the process of PD-L1 expression in tumors. Experiments in vitro demonstrated that IL-6 enhanced PD-L1 expression in the tumor tissue through the JAK1/Stat3 pathway, leading to immune evasion [95].

Lymphocytes such as CD8+ T cells and Treg cells also participate in this process. DENG H, et al. identified a tumor-reactive tumor-infiltrating T lymphocyte (TIL) pool, termed PD-1T TILs, which have predictive potential in advanced NSCLC patients treated with PD-1 blockade. High PD-1T TILs were associated with significantly longer PFS (HR = 0.39, 95% CI: 0.24–0.63, p<0.0001) and OS (HR = 0.46, 95% CI: 0.28–0.76, p<0.01). Moreover, these TILs effectively identified patients who would not benefit from ICIs, indicating their high negative predictive value (NPV) [96]. Tumor infiltration was also a biomarker for immunotherapy combined with chemotherapy, revealed by analysis of the ORIENT-11 study [79]. This can assist clinicians in easily identifying a patient group without benefit.

### 4.2. Circulating Fatty Acid Profile

Lipid metabolism has been demonstrated to play a crucial role in the regulation of immune functions [97]. Specifically, tumor tissues exhibit abnormal activation of de novo lipogenesis due to the overexpression of fatty acid synthase, ATP citrate lyase, and acetyl-CoA carboxylase [98]. This dysregulation has been associated with an unfavorable outcome in cancer patients. The upregulation of adipogenesis promotes cancer cell proliferation by providing a continuous supply of substrates for membrane formation and bioenergy production [99]. Therefore, lipid mediators have the potential to serve as biomarkers for individual sensitivity to ICIs. GALLI G, et al. discovered that certain esterified medium chain (C18:0) and unsaturated (C16:1) fatty acids were positively correlated with prognosis following immunotherapy. Conversely, an esterified saturated fatty acid (C16:0) was found to be associated with a poorer outcome in NSCLC patients treated with ICIs [100]. Previous research has shown that lipid metabolic signaling plays a significant role in TME and immunotherapy [101]. Additionally, high-mutated lipid metabolism signaling was associated with prolonged PFS in NSCLC patients who receive ICIs, due to enhanced immunogenicity. Moreover, patients with a higher number of mutations exhibited significantly TMB and PD-L1 expression [102]. Therefore, hypermutated lipid metabolism signaling has the potential to serve as a biomarker for efficacy of ICIs in NSCLC. In summary, further investigation is needed to explore the correlation between lipid metabolism and the efficacy of immunotherapy.

### 4.3. Microbiome

Microbiome, as a hallmark of cancer, plays a crucial role in anti-cancer immunity [103,104]. Specifically, it influences the efficacy of ICI treatments in various tumor types [105,106,107,108,109]. Clinical evidence has shown that antibiotics have a detrimental impact on the clinical benefits of immunotherapy [104,105,106,107,108,109,110]. A retrospective study (*N* = 65) found that responders to ICIs exhibit a distinct microbiome structure, characterized by an enrichment in amplicon sequence variants (ASVs) belonging to the genera *Ruminococcus*, *Akkermansia*, and *Faecalibacterium* [109]. To elucidate this correlation, Routy et al. looked into patients and mice and revealed an association between a higher richness of gut microbiota and a better clinical response to PD-1 inhibitors [110]. A. *muciniphila* was found to play a crucial role in this response. Meanwhile, B. *fragilis* was found to significantly impact the gut microbiota in anti-CTLA-4 treatment [111]. Other clinical cohorts have also confirmed the role of gut microbiota as biomarkers for ICIs. One retrospective study (*N* = 11) identified ketones and alkanes as risk factors for early progression and short chain fatty acids (SCFAs), such as propionate and butyrate, as biomarkers for long-term beneficial effects [112]. The PEOPLE study, a prospective phase II trial (*N* = 65), discovered a correlation between E. *massiliensis* and PFS in patients with advanced NSCLC who had PD-L1 levels below 50% [113]. Notably, this correlation may be mediated by the tumor microenvironment. In mice, probiotic supplementation induced the upregulation of SCFAs in the gut and blood, promoting the recruitment of Th17 cells and attenuation of lung metastasis [114]. However, the exact mechanism underlying this process is still being studied. Furthermore, it has been found that the gut-microbe-derived metabolite trimethylamine N-oxide (TMAO) enhances antitumor immunity in pancreatic ductal adenocarcinoma, mediating the improved efficacy of ICIs [115].

Apart from gut microbiome, infection in the gastrointestinal tract can also affect the efficacy of ICIs. H. *pylori* infection has been associated with a poorer response to anti-PD1 treatment in patients with NSCLC (6.7 months vs. 15.4 months, *p* = 0.001), observed in mice and patients in a retrospective study (*N* = 89). In vitro and in vivo experiments have demonstrated that dendritic cells mediate a reduced proliferation and activation of CD8+ T cells in the presence of H. pylori infection [116]. Additionally, other potential microbiota biomarkers, including the lower airway microbiome that shapes host immune tone [117] and the oral microbiome that has been correlated with lung cancer risks in never smokers [103], await further examination. These biomarkers have the potential to serve as indicators of immunotherapy efficacy.

## 5. Discussion

Recent advancements in precision medicine have significantly accelerated immunotherapy research, particularly in the context of NSCLC. While immunotherapy shows promise as a treatment strategy, a substantial number of patients still struggle to benefit from it. Identifying the factors that determine which driver-gene-negative advanced NSCLC patients will respond favorably to ICI treatment remains an ongoing challenge. As summarized in the Cancer-Immunity Cycle [118], a series of events would affect the anticancer immune response. From the release of cancer cell antigens, T-cell activation and infiltration, to the recognition and killing of cancer cells, each of these procedures would impact the ultimate efficacy of immunotherapy. PD-1/L1 inhibitors are key effectors in the priming and activation step and the killing step. In several trials [9,10,11,12,13], PD-L1 expression have been proved to be closely related to the efficacy of immunotherapy, and further associated with the clinical outcomes. However, there also are studies [21,22,23,24,25,26] that failed to observe these associations, especially for trials where immunotherapy were combined with chemotherapy [4,16,18,19,20]. Similarly, tumor mutation burden were found to be predictive for immunotherapy. It may serve as a biomarker, possibly correlating with the presence of neoantigens and indicating the neoantigen load of the tumor. However, there may be a nonlinear relationship between TMB expression levels and curability. In immune monotherapy, for example, the FDA has confirmed that in the treatment of solid tumors, populations with high levels of TMB are more likely to benefit from pembrolizumab [119]. However, in the combination of chemotherapy and immunotherapy, TMB and PD-L1 have similar characteristics, showing that its predictive performance is reduced [61,62]. As far as TME-related factors and other host-related factors are concerned, there are still difficulties in the detection methods, and because they are more influenced by host factors, their predictive performance for prognosis is not accurate. There have been positive results in some clinical trials for the predictive effect of specific gene mutations on prognosis in immunotherapy [37,73,120]. However, the data come from a single clinical trial cohort and their correlation with prognosis needs to be further investigated after integration of data from multiple clinical trials. In summary, we can see that the forecasting power of a single indicator is limited. The future direction of development is to establish a joint forecasting model.

## 6. Conclusions

Immunotherapy significantly improves the prognosis of driver-gene-negative NSCLC patients, regardless of the level of PD-1/L1 expression in the patients. Although PD-1/L1 expression is an important predictor of whether an immunotherapy will be of benefit to the population, its expression level alone is not enough. It seems that combining PD-L1, TMB, TME markers, pathway abnormalities, and host factors to create a multi-dimensional biomarker efficacy prediction model is the way to go.

## Figures and Tables

**Table 1:** Summary of HR for mOS or mPFS based on PD-L1 expression in first-line immuno-monotherapy.

Clinical Trial	Pathological Type	Treatment Arms	HR for mOS (95% CI) by PD-L1 (%)	HR for PFS (or mPFS) (95% CI) by PD-L1 (%)
CheckMate-026 [14]	NSCLC (PD-L1 ≥ 1%)	Nivolumab vs. chemotherapy	≥5% HR = 1.02, 95% CI 0.80–1.30	≥5% HR = 1.15, 95% CI 0.91–1.45
MYSTIC [15]	NSCLC	Durvalumab vs. chemotherapy	TC ≥ 25% HR = 0.76; 97.54% CI 0.56–1.02	
KEYNOTE-024 [10]	NSCLC (PD-L1 TPS ≥ 50% and EGFR/ALK WT)	Pembrolizumab vs. chemotherapy	≥50% HR = 0.62, 95% CI 0.48–0.81	
KEYNOTE-042 [11]	NSCLC (PD-L1 TPS ≥ 1% and EGFR/ALK WT)	Pembrolizumab vs. chemotherapy	≥50% HR = 0.69, 95% CI 0.56–0.85	
			1–49% HR = 0.92, 95% CI 0.77–1.11	
			≥1% HR = 0.81, 95% CI 0.71–0.93	
IMpower110 [12]	NSCLC	Atezolizumab vs. chemotherapy	TC/IC 3 HR = 0.59, 95% CI 0.4–0.89	
			TC/IC 2/3 HR = 0.72, 95% CI 0.52–0.99	
			TC/IC 1/2/3 HR = 0.83, 95% CI 0.65–1.07	
			TC 1/2 HR = 1.04, 95% CI 0.76–1.44	
EMPOWER-Lung 1 [13]	NSCLC (PD-L1 TPS ≥ 50% and EGFR/ALK/ROS1 WT)	Cemiplimab vs. chemotherapy	≥50% HR = 0.57, 95% CI 0.42–0.77	

Note: CI, confidence interval; HR, hazard ratio; IC, immune cell; ITT, intention to treat; NA, not available; NR, not reached; ORR, objective response rate; mOS, median overall survival; mPFS, median progression free survival; TC, tumor cell; TC/IC 0, PD-L1 expression on 0% of tumor and immune cells; TC/IC 1, PD-L1 expression of ≥1% on either tumor cells or immune cells; TC/IC 2, PD-L1 expression on 1–49% of tumor cells or on 1–10% of immune cells; TC/IC 3, PD-L1 expression on ≥50% of tumor cells or on ≥10% of immune cells; WT, wild type.

## Abbreviations

The following abbreviations are used in this manuscript:

ASVs	Amplicon Sequence Variants
bTMB	Blood-Tumor Mutation Burden
CI	Confidence Interval
CRP	C-reactive Protein
CT	Chemotherapy
D	Durvalumab
EVs	Extracellular Vesicles
HR	Hazard Ratio
IC	Immune Cell
ICIs	Immune checkpoint inhibitors
ITT	Intention-to-treat
KEAP1	Kelch-like ECH-associated Protein 1
LC	Lymphocyte Count
LDH	Lactate Dehydrogenase
LIPI	Lung Immune Prognostic Index
mOS	Median Overall Survival
mPFS	Median Progression-Free Survival
NA	Not Available
NC	Neutrophil Count
NCCN	National Comprehensive Cancer Network
NE	Not Estimable
NLR	Neutrophil-to-lymphocyte Ratio
NPV	Negative Predictive Value
NRF2	Nuclear Factor Erythroid 2-related Factor 2
NSCLC	Non-Small Cell Lung Cancer
NSQ NSCLC	Non-Squamous Non-Small Cell Lung Cancer
OS	Overall Survival
PFS	Progression-Free Survival
PLR	Platelet-to-Lymphocyte Ratio
SCFAs	Short-Chain Fatty Acids
SIPS	Scottish Inflammatory Prognostic Score
SQ NSCLC	Squamous Non-Small Cell Lung Cancer
STK11	Serine/Threonine Kinase 11
T	Tremelimumab
TC	Tumor Cell
TCR	T-cell Receptors
TGF-β	Transforming Growth Factor-β
TIL	Tumor-iInfiltrating T Lymphocyte
TMAO	Trimethylamine N-Oxide
TMB	Tumor Mutation Burden
TME	Tumor Immune Microenvironment
TPS	Tumor Proportion Score
tTMB	Tissue-TMB
WCC	White Cell Count
WT	Wild Type
